# Optically Remote Control of Miniaturized 3D Reconfigurable CRLH Printed Self-Powered MIMO Antenna Array for 5G Applications

**DOI:** 10.3390/mi13122061

**Published:** 2022-11-24

**Authors:** Hayder H. Al-khaylani, Taha A. Elwi, Abdullahi A. Ibrahim

**Affiliations:** 1 Electrical and Computer Engineering Department, Faculty of Engineering, Altinbas University, Istanbul 34217, Turkey; 2 Department of Communication Engineering, Al-Ma’moon University College, Baghdad 1004, Iraq; 3 International Applied and Theoretical Research Center (IATRC), Baghdad 10001, Iraq

**Keywords:** optical switching, beam steering, CRLH, LDR, 5G

## Abstract

A novel design of a reconfigurable MIMO antenna array of a 3D geometry-based solar cell integration that is operating at sub-6 GHz for self-power applications in a 5G modern wireless communication network. The proposed antenna array provides three main frequency bands around 3.6 GHz, 3.9 GHz, and 4.9 GHz, with excellent matching impedance of S_11_ ≤ −10 dB. The proposed MIMO array is constructed from four antenna elements arranged on a cubical structure to provide a low mutual coupling, below −20 dB, over all frequency bands of interest. Each antenna element is excited with a coplanar waveguide (CPW). The proposed radiation patterns are controlled with two optical switches of Light Dependent Resistors (LDRs). The proposed antenna array is fabricated and tested experimentally in terms of S-parameters, gain and radiation patterns. The maximum gain is found to be 3.6 dBi, 6.9 dBi, and 3.5 dBi at 3.6 GHz, 3.9 GHz, and 4.9 GHz, respectively. It is realized that the proposed array realizes a significant beam forming by splitting the antenna beam and changing the main lobe direction at 3.9 GHz after changing LDR switching statuses. Such an antenna array is found to be very applicable for femtocell wireless communication networks in the 5G systems.

## 1. Introduction

Fifth generation (5G) is a developed technology of mobile broadband communication systems [1]. The frequency spectrum of 5G systems is used in two ranges, sub-6 GHz and mmW bands [2]. Different 5G antennas were proposed for 5G specifications [3]-based single elements and arrays systems. The authors in [4] attempted to produce an exhaustive review to cover the main important antenna design specifications including gain, bandwidth, and radiation patterns [5]. Later, different studies applied the concepts of composite right/left hand (CRLH) metamaterials in their designs due to their unusual electromagnetic properties [6]. Such antennas realized unique properties with exceptional devices for the most urgent applications [7]. Therefore, CRLH structures were introduced to different antennas to realize performance enhancement, as in [8]. A multiband fractal antenna was utilized based on circler and triangular shapes to operate within the sub-6 GHz bands [9]. The influence of introducing Koch fractal geometry on the edges of a square microstrip patch antenna is to enhance the antenna bandwidth by reducing the surface wave effects [10]. In [11], a compact antenna array with eight elements of loop structure was utilized for 5G mobile devices at sub-6 GHz. Another design was proposed in [12] for 5G systems with 10 elements at sub-6 GHz ranges. Each individual antenna element is designed with a T-shaped slot patch fed with a T-shaped line. Finally, a design of an antenna array with 18 elements was developed in [13] for MIMO 5G portable communication systems smartphones at sub-6 GHz bands.

In another concept, the introduction of the solar panel to the modern antenna designs has become the most urgent for the self-powered wireless systems [14]. For this, the MTM-based antennas were integrated with solar panels in different publications, and the authors in [15] proposed an antenna design based on MTM for gain enhancements by integration of their antenna design with the solar panel as a substrate. In [16], a design of the MTM structure was based on a plasmonic antenna array for modern applications; in that design, the authors printed their antenna array on a flexible solar panel. Another design of an antenna-based MTM was introduced for self-powered wireless systems by attaching the antenna ground plane to the solar panel [17]. The main novelty of this work is introducing the use of the solar panel as a perpendicular addition to the antenna array in the aim of reducing the effects of grating lobs and conducting the green energy for self-powered wireless systems. Such an introduction of the solar panel has no negative effects on the antenna array performances. Nevertheless, the antenna array performance is controlled by LDR switches without the need for DC biasing circuits.

This paper is organized as follows: In Section 2, the antenna array geometric details are presented with all relative geometrical dimensions. Unit cell effects are discussed in Section 3, Section 4 presents the Design Methodology. The theoretical results and discussions are shown in Section 5. In Section 6, the Results validations are clarified. Section 7 shows the solar panel effects and, finally, the conclusion of this work is presented in Section 8.

## 2. Geometrical Details

**Figure 1:**
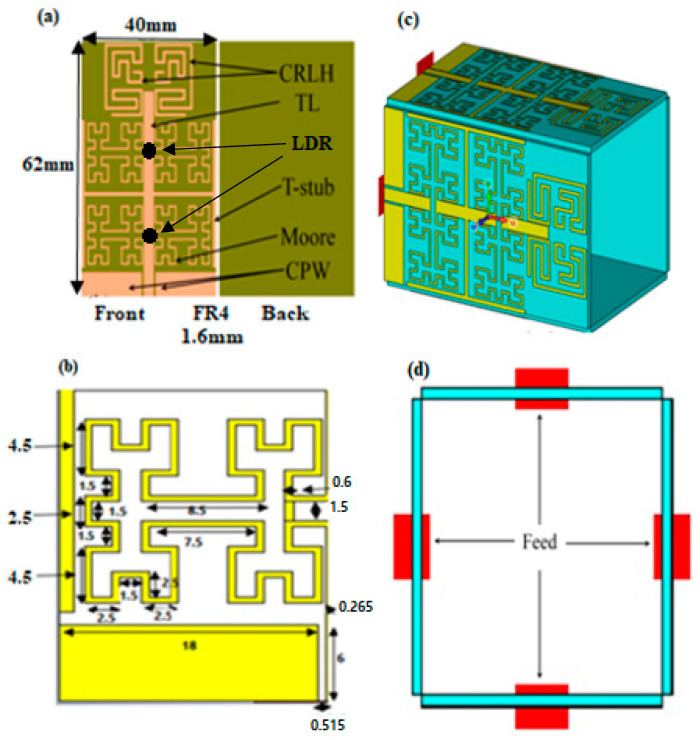
Antenna geometrical details: (**a**) Single-antenna elements, (**b**) one Moore fractal, (**c**) MIMO array 3D view, and (**d**) MIMO array top view. All dimensions are in mm scale.

The proposed antenna design is based on four Moore fractal MTM unit cells with a coplanar waveguide feed, microstrip line, matching load circuits, and T-stubs, as seen in Figure 1a. Four Moore fractal unit cells have been utilized in the proposed antenna to increase the antenna size reduction and realize multiband resonances. The antenna is printed on an FR4 Techtronic epoxy substrate with dimensions of 62 × 40 mm^2^. A bandwidth enhancement can be achieved within a limited area by magnifying a surface current that is afforded on an electrically long path. The coplanar waveguide is used to avoid the capacitive coupling between the antenna patch and the ground plane [18]. Nevertheless, such a design maintains the other surface of the antenna substrate without any printed circuit to be used for busing electronic circuit. The transmission line is designed with the width of 0.6 to realize a 50 Ω matching circuit and to transfer the surface current motion to the proposed Moore structure. The gap on the transmission line is introduced to force the current motion toward the fractal geometry. The proposed T-stub is introduced to suppress the surface waves from the antenna edges that negatively affect the radiation efficiency [18,19]. The proposed CRLH inclusions are introduced to avoid surface wave retardation that could be created in an opposite interference and inductive loss due to the use of the transmission line junction that can be removed by the capacitive effects of the matching circuit. Therefore, a significant suppressing could be achieved in the surface wave at the substrate edges to avoid coupling effects with other antenna elements. The CRLH used in this study is to enhance the antenna bandwidth and reduce the antenna size as well as increasing the antenna gain by suppressing the surface on the edges of the element [20]. The microstrip patch is printed on an FR4 substrate without a ground plane. The substrate dielectric constant is 4.3 with a height of 1.67 mm. Two photo resistors are used in the antenna structure to obtain different operating frequencies and radiation patterns by switching the Light Dependent Resistors ON/OFF.

The proposed array is consistent of four antenna elements mounted on an epoxy FR4 substrate of an area of 62 × 40 mm^2^ to form a cubical geometry. The proposed work is based on a self-decoupling technique by configuring the antenna array on a 3D structure. Such a configuration maintains the ability of decoupling between the antenna elements if they are arranged perpendicular to each other [21].

**Figure 2:**
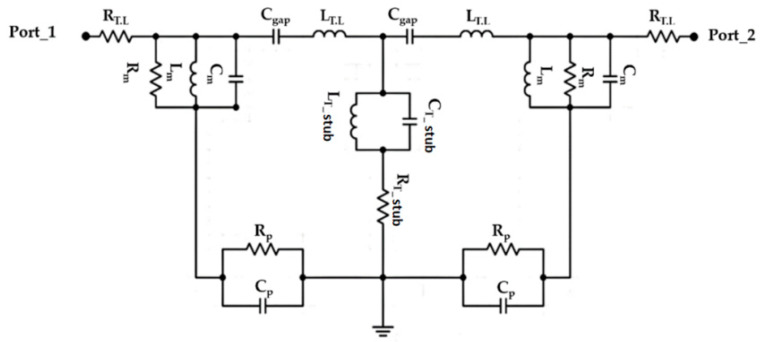
Equivalent circuit model.

MTM structures are defined according to the electromagnetic properties. It can be designed accurately to gain a negative refractive index at certain frequency bands. For this reason, these materials depend on RH and LH components [21]. Therefore, the combination of the open T-stub with CRLH and the antenna circuitry can be discussed using an equivalent circuit model that is shown in Figure 2. Therefore, the proposed structure operation is described using the Richard model [3]; that is, based on the lumped element theory [8]. It is good to mention that the proposed Moore geometry dimensions are calculated to obtain a resonance within the frequency band from 3 GHz to 6 GHz. The proposed structure-based Moore shape can be described with a fractal segment, which can be determined [2] as follows:

(1) $$N_{n}={\mathrm{nN}}_{1}$$

where n is the fractal iteration, N_1_ is the fractal segment number, and N_n_ is the fractal order. This revealed that the total fractal length $L^{n}$ can be calculated as:

(2) $$L^{n}=\frac{8n}{2n+3}L_{0}^{n}$$

where $L_{0}^{n}$ is the perimeter of a conventional rectangle that is occupied by the fractal. Therefore, the length of the total perimeter increases with the external side edge increase as follows:

(3) $$L\left(n\right)=\left(2^{n}+1\right)S$$

From Equation (3), the perimeter is found to increase exponentially with n increase. The proposed antenna geometry based on the open T-stub and the 4th order of Moore geometry is analyzed analytically using an equivalent circuit model based on the lumped elements Richard model [5]. As seen in Figure 2, the proposed circuit model is considered by connecting a 50 Ω input impedance RF source in series with an (R-L-C) parallel branch to be named as L_m_ = 1.1 nH, R_m_ = 21 Ω, and C_m_ = 0.1 pF for the Moore structure. The main transmission line is characterized by an inductive part L_T_ = 2 nH and capacitive air gaps C_gap_ = 1.3 pF. The open T-stub as a load is connected to the center of the transmission line and defined as L_T-stub_ = 1.6 nH in parallel with a capacitor of C_T-stub_ = 2.3 pF. This branch is connected serially with a resistor of R_T-stub_ = 30 Ω. Each of R_p_ = 22 Ω and C_p_ = 2.3 pF are connected in parallel with the equivalent circuit model to denote the pans effects. The lumped elements of the presented circuit in Figure 2 are evaluated from the Advanced Design System (ADS) software package.

## 3. Unit Cell Effects

**Figure 3:**
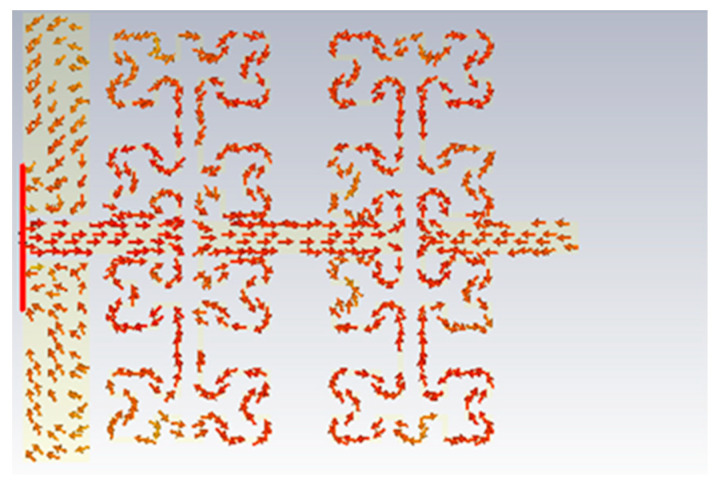
Current motion on the proposed Moore unit cells.

The proposed antenna design is mainly structured from four Moore fractal geometry cells. Two Moore cells are created in a proposed antenna substrate (each two adjacent Moore fractal horizontally represent one Moore cell). So, in order to study the effects of them on the antenna performance, the authors utilized a parametric study based on (CST MWS). In simulation, the authors added the first Moore fractal unit cell to the proposed antenna design, then compared the gained results with the identical antenna, which was designed by using two Moore cells. As we found in the first scenario, the antenna displays multiple frequency bands within the range of interest frequency. However, after introducing the other Moore fractal unit cell, the proposed antenna produced varying frequency bands within the interest frequency range. This is realized due to the type of connection between the two-unit cells on the transmission line series. In series connection, the direction of current motion is mostly selfsame of two-unit cells, where the combination in series connection led to adding the effects of them to each other directly. Each Moore unit cell is a capacitive inclusion, where the increasing of their number is to achieve the significant equivalent capacitor reduction [20], which leads to an increase in the particular operating frequency [22]. Figure 3 shows the surface current motion on Moore unit cells in the proposed antenna.

**Figure 4:**
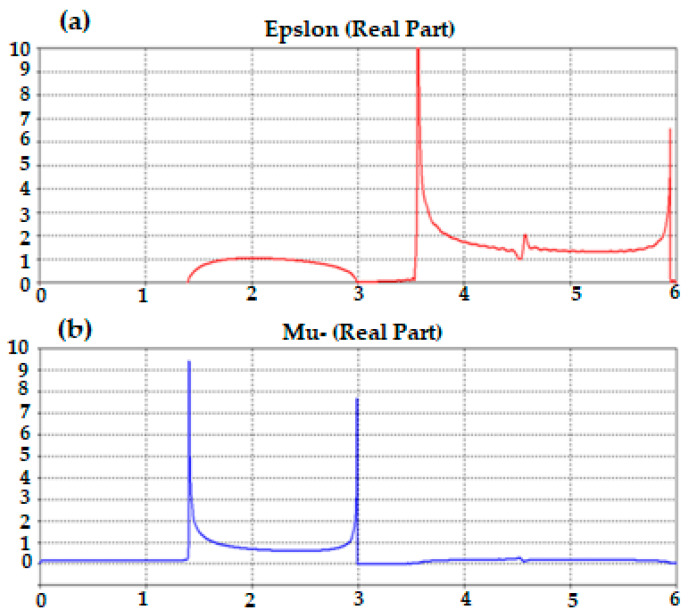
Moore unit cell electromagnetic properties: (**a**) Permittivity and (**b**) Permeability.

Next, the authors clarified in Figure 4 the relative electromagnetic properties, in terms of permittivity and permeability of the proposed Moore unit cell, where it is found that the proposed Moore unit cell provides near-zero permittivity and permeability within the interest frequency band. This observation displays that the proposed Moore unit cell is a stellar candidate to grow the surface current; this is by reactive parts reduction of the antenna patch to the antenna bandwidth increasing [20].

## 4. Design Methodology

A. Single Antenna Design

**Figure 5:**
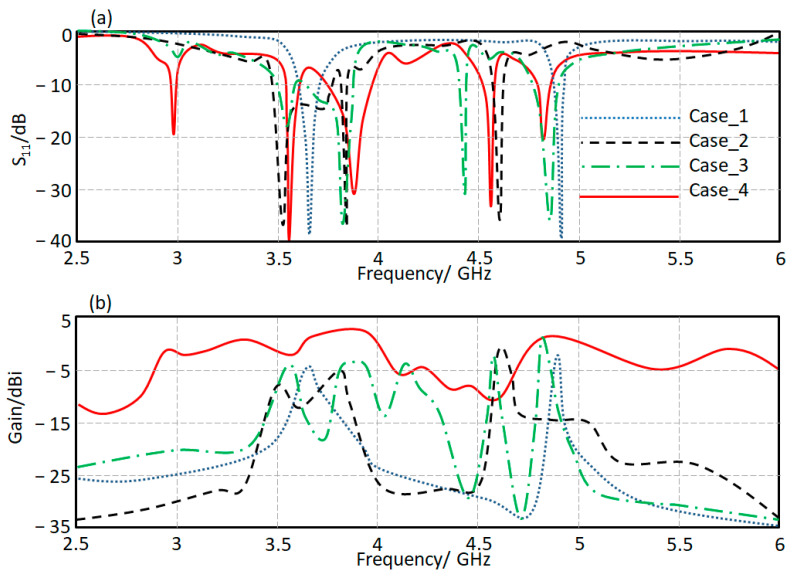
Antenna performance based on a single antenna element: (**a**) S_11_ and (**b**) Gain.

The proposed antenna structure consists of four main parts. The first one is a monopole transmission printed line circuit (case_1). The second case (case_2) is based on the T-stub structure with the transmission line circuit. The introduction of the Moore structures to the proposed design is called case_3. Finally, the proposed antenna structure in Figure 1a is presented in case_4 after the matching circuit introduction. The proposed cases’ performances are calculated numerically using CST MWS in terms of S_11_ and gain spectra, as shown in Figure 5a,b. Based on the evaluated results, it is found that the proposed antenna bandwidth is enhanced significantly after introducing our matching circuit to design in case_4 with a maximum gain of 4.5 dBi at 3.9 GHz and 4.9 GHz. However, the first mode is generated, at 2.96 GHz, from the Moore fractal structure introduction. The other two modes were generated from the proposed designs in case_2 and case_3. The fundamental modes are generated by case_1 due to the current motion in the fractal geometry [22].

B. 3D array Design

**Figure 6:**
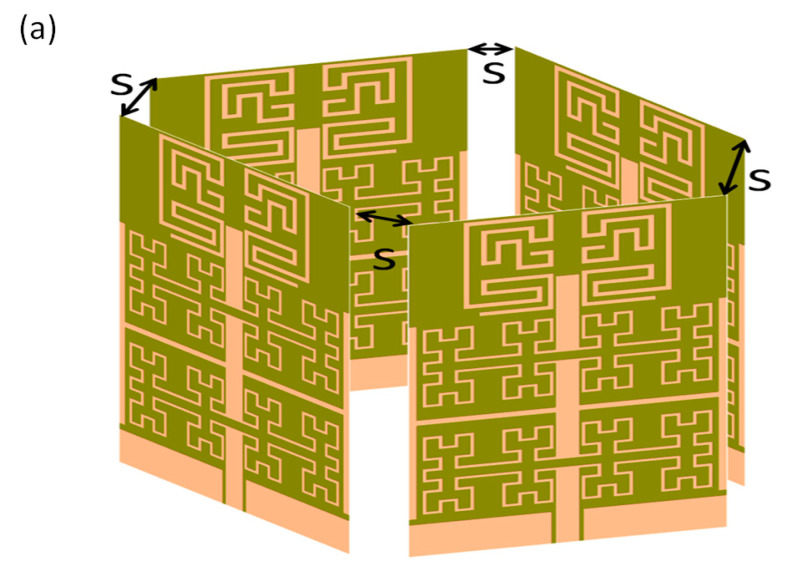

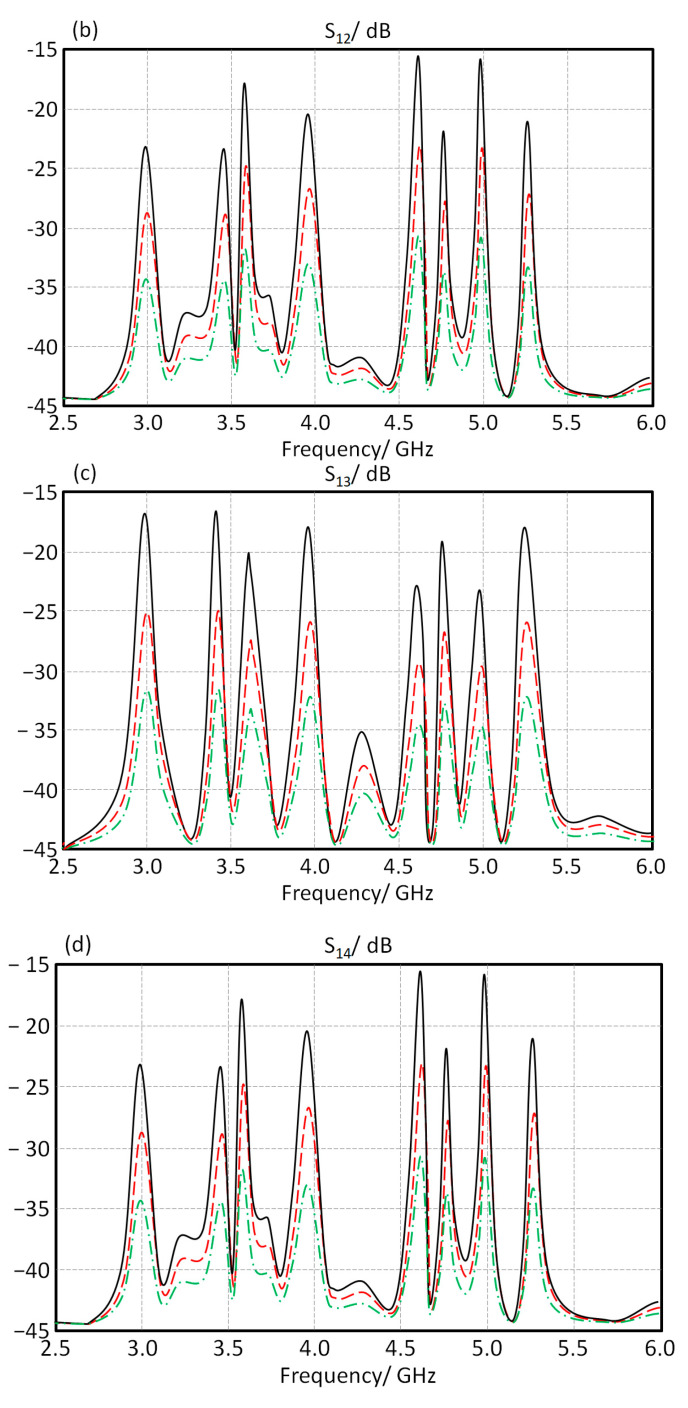
Coupling effects of 3D array configuration in terms of: (**a**) Antenna array model (**b**) S_12_, (**c**) S_13_, and (**d**) S_14_ spectra. Note: The solid black line is for S = 3 mm, the discrete red for S = 6 mm, and the discrete green line for S = 9 mm.

The proposed antenna array is formed in the shape of a 3D configuration, as shown in Figure 6a. Therefore, the proposed configuration with different separation distance(s) to monitor the mutual coupling between the antenna elements, S_12_, S_13_, S_14_, is shown in Figure 6b–d. The parameter is changed from 3 to 9 mm with a step 3 mm. It is recognized when S = 3 mm; the coupling in general is less than −15 dB. However, when S = 6 mm, the coupling is reduced to less than −23 dB. After reaching S = 9 mm, the mutual coupling becomes much less than −30 dB for S_12_, S_13_, and S_14_ spectra at the frequency band of interest. Therefore, the authors considered that S = 6 mm is enough for the design specification to provide mutual coupling of −20 dB.

## 5. Theoretical Results and Discussion

**Figure 7:**
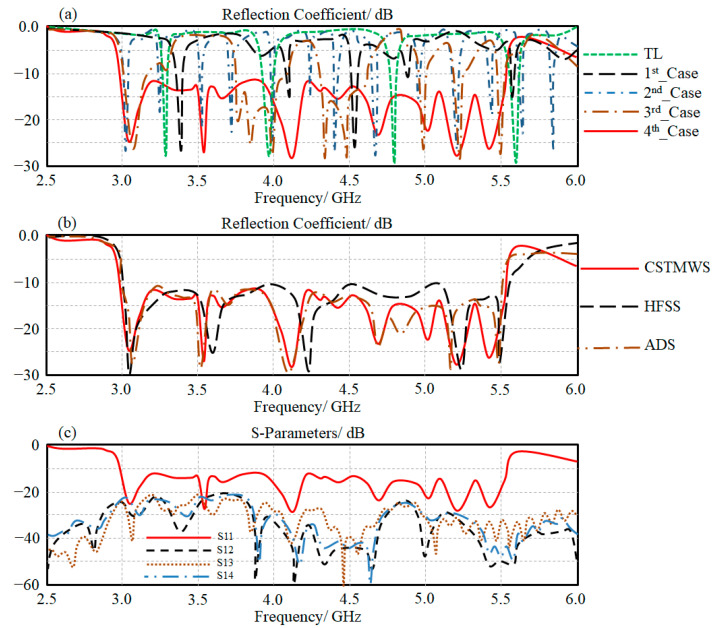
S-parameters spectra evaluation: (**a**) reflection coefficient (dB) of the considered cases, (**b**) validated reflection coefficient (dB) results, and (**c**) S-parameters (dB): S_11_, S_12_, S_13_, S_14_.

In this section, a theoretical study based on a numerical simulation is conducted first. In this part, a parametric study using CSTMWS is invoked to realize the influence of introducing each antenna part to the design separately as follows. The 1st_case is considered the transmission line with air gaps. For the 2nd_case, the Moore fractal is introduced to the antenna. For the 3rd_case, the open T-stubs are introduced to the design. The effects of the proposed CRLH inclusions are realized for the 4th_case. This study is invoked to evaluate the effects of each part introduction on the antenna S_11_ spectra to be compared to those obtained from ADS. The proposed antenna element performance based on the considered cases is evaluated in terms of S_11_ spectra using CST MWS, as seen in Figure 7a. The effects on introducing the open T-stub and CRLH structures on the antenna bandwidth enhancement are obvious. Later, the obtained results are revaluated using HFSS and ADS for validation, as seen in Figure 7b. It is found that the simulated results agree to each other excellently.

In terms of an antenna MIMO performance-based antenna array with four elements, the proposed array is structured inside a CST MWS environment as a cubical array. The array performance is described with S-parameters in terms of S_11_, S_12_, S_13_, and S_14_ spectra, as seen in Figure 7c. For this, the authors applied this to validate the ability of the proposed technique to reduce the mutual coupling between the antenna elements. Figure 7c shows that the maximum coupling between adjacent elements is below −20 dB.

The switching process is controlled through illuminating the LDR structures with light to act as an ON/OFF status. The use of the LDR structures realizes a surface current control that results in a significant change in the guided wavelength that changes the antenna radiation to the desired location [3]. Therefore, the effects of changing the antenna radiation patterns in terms of the main lobe direction are listed in Table 1. It is observed that, when the LDR are switched to OFF:OFF and OFF:ON status, the main lobe is devoted away from the feeding structure. Whereas, when switching the LDR to ON:OFF and ON:ON, the main lobe is devoted toward the feed structure.

## 6. Results Validation

**Figure 8:**
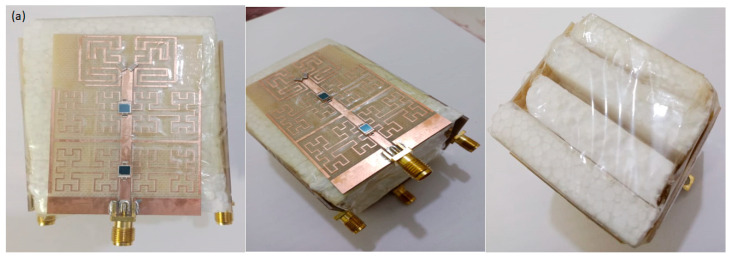

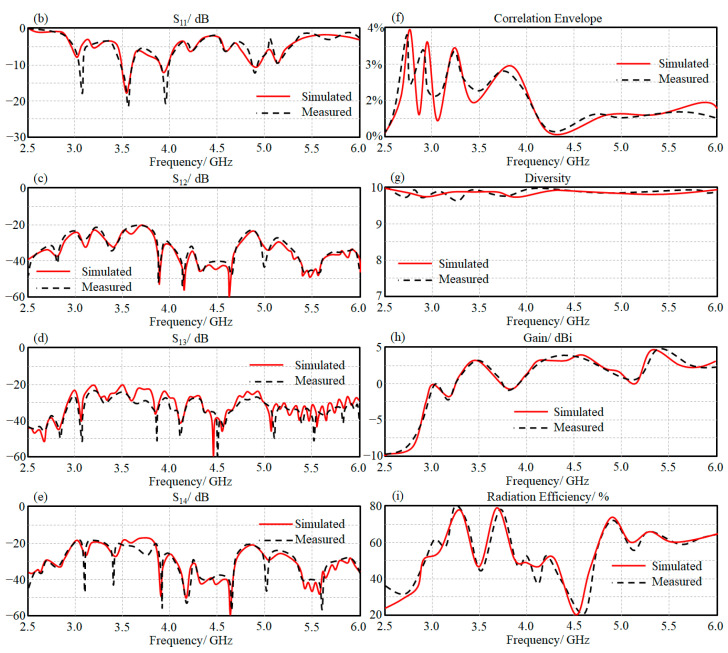
Experimental work: (**a**) fabricated prototype, (**b**) S_11_, (**c**) S_12_, (**d**) S_13_, (**e**) S_14_, (**f**) correlation envelop, (**g**) diversity envelope, (**h**) gain spectra, and (**i**) radiation efficiency.

In this section, the proposed antenna array performance is measured using a vector network analyzer (37347A) and RF chamber, as below:

The prototype of the MIMO antenna is fabricated using FR4 substrate, as shown in Figure 8a. The proposed antenna array performance is controlled using LDR switches that acquire no biasing systems for operation. In such a case, the novelty with this design is how to ensure the surface current motion on the patch through LDR switches.

Now, the antenna measurements in terms of S-parameters are presented when all LDR are ON, as shown in Figure 8b–e, where the S_11_ spectrum represents the return loss, S_11_ ≤ −10 dB for the frequency band of interest. S_12_ and S_14_ spectra represent the mutual coupling between adjacent elements which must be ≤ −20 dB and the mutual coupling between the radial element (between the opposite side elements) is S_13_. It is found from the measured result in the black curve that there is an excellent agreement with the red curve (simulated) with insignificant discrepancy.

**Figure 9:**
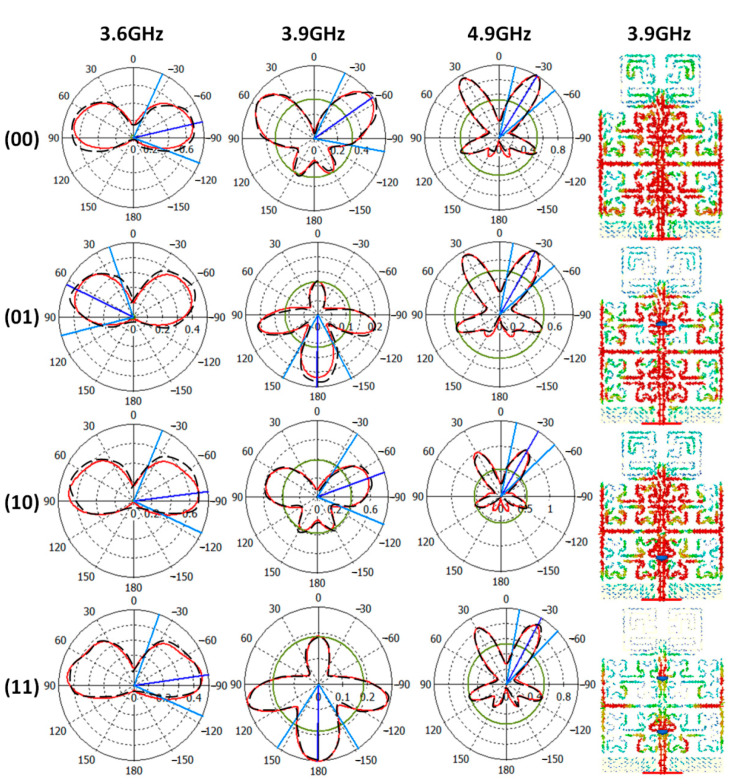
Measured radiation patterns at 3.6 GHz, 3.9 GHz, and 4.9 GHz and current distributions at 3.9 GHz only. Note: The black denoted line is for measurements and the red solid line for simulated results. In addition, the current distributions are from simulation only.

Next, the measured and simulated radiation patterns at 3.6 GHz, 3.9 GHz, and 4.9 GHz are shown in Figure 9. The radiation pattern measurements are done inside an RF anechoic chamber. For these measurements, the authors conducted the measurements after switching the LDR structure ON and OFF by illuminating them with a laser diode.

To explain the reconfiguration mechanism, the surface current distributions at 3.9 GHz only is explained in Figure 9. It is found that, at 3.9 GHz specifically, the optical switches, LDR, realize a significant effect through controlling the antenna surface current, as seen in Figure 9. Nevertheless, the main lobe of the proposed antenna is found to be split into two beams at the frequency band of interest. Such a phenomenon is attributed to the effects of the T-stub introduction [7]. However, using the proposed switches performs further forming on the main lobes such as directing the antenna radiation to the opposite direction, as shown in Figure 9 at 3.9 GHz. This is achieved by controlling the surface current motion on the antenna patch. In this work, LDR switch loads are introduced to increase the surface current magnitude to a certain direction [4]. In such a case, the antenna portion with less resistance mitigates the electromagnetic energy flow from the other antenna parts.

Later, the measured and simulated radiation patterns at 3.6 GHz, 3.9 GHz, and 4.9 GHz are shown in Figure 9. The radiation pattern measurements are done inside an RF anechoic chamber. For these measurements, the authors conducted the measurements after switching LDRs to ON and/or OFF according to 00, 01, 10, and 11 by illuminating them with a laser diode.

**Figure 10:**
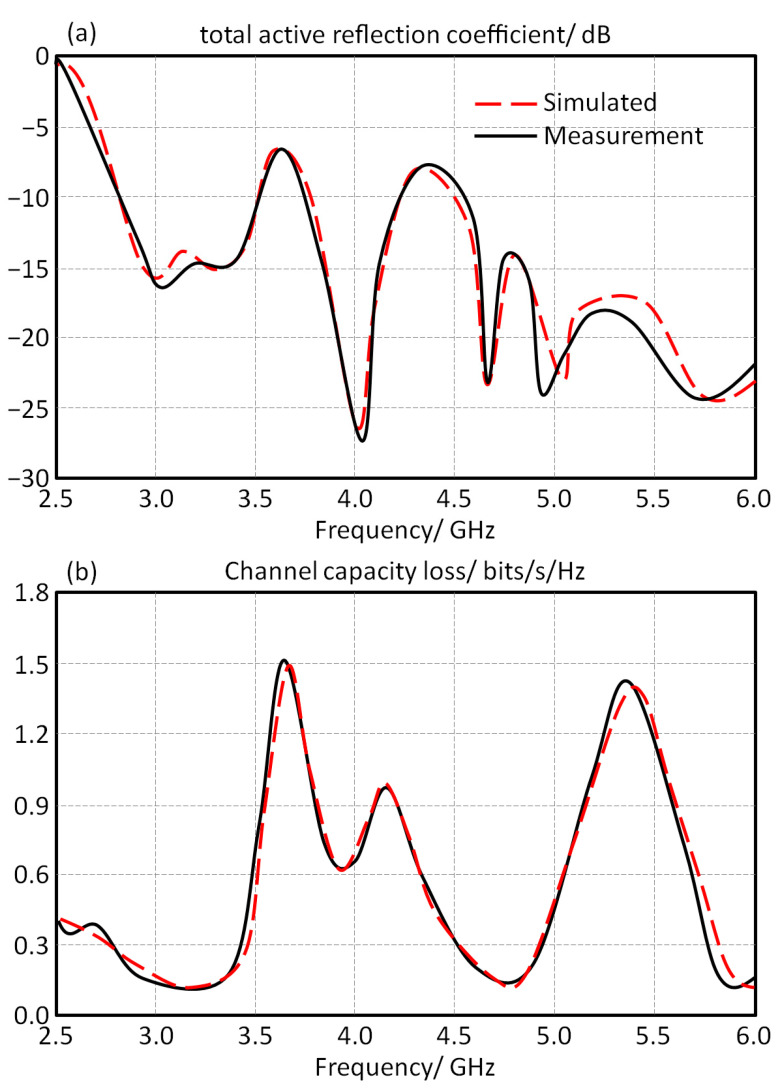
Antenna array performance in terms of channel response: (**a**) total active reflection coefficient and (**b**) channel capacity loss.

Figure 10a shows the total active reflection coupling between all the exciting ports, which refers to the effective BW of MIMO systems. We can observe that the maximum value of the total active reflection coupling is below −15 dB at the frequency bands of interest.

The measured and simulated values of channel capacity losses are measured to be compared against the simulated results in Figure 10b. It is found that the obtained values are less than 0.4 bits/s/Hz with the operating bands. Additionally, the proposed antenna MIMO array is compared to the previously published works, as listed in Table 2.

Finally, it is concluded after comparing the proposed antenna performance to the published results in the literature that the proposed antenna array provides two advantages over other published results. The first one in terms of radiation patterns forming can be controlled optically using LDR switches. Regarding the second advantage, it is found that the proposed switching technique acquires no wiring system or external biasing circuit to control the antenna performance. Therefore, such a facility in the proposed antenna array recognizes a remarkable technology over all the previous studies. Also, it is found that the proposed antenna array provides a frequency bandwidth from 3.1 GHz to 5.75 GHz with a matching impedance of S_11_ ≤ −10 dB based on 3D array configuration.

## 7. Solar Panel Effects

A. Antenna Performance

**Figure 11:**
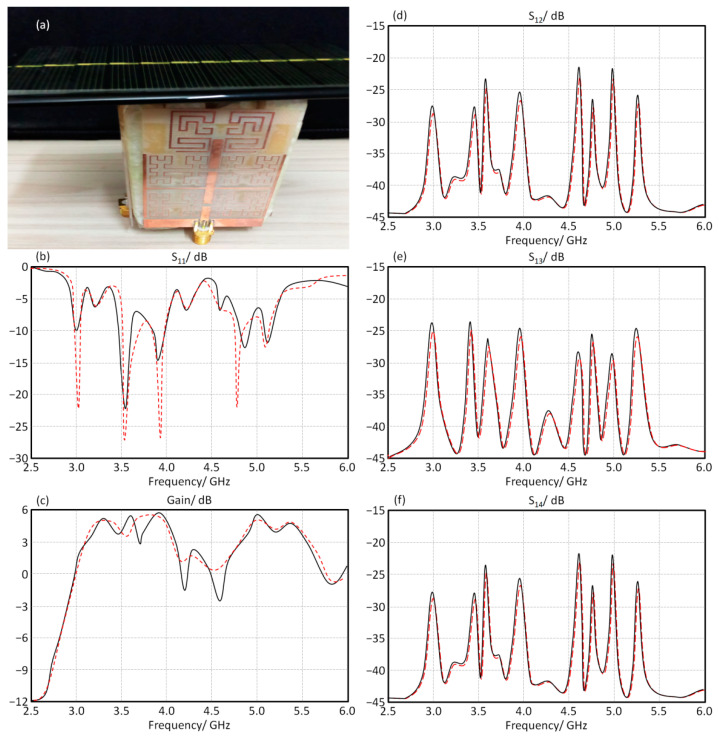
Antenna performance with solar panel introduction; (**a**) antenna design, (**b**) S_11_, (**c**) gain, (**d**) S_12_, (**e**) S_13_, and (**f**) S_14_ spectra. Note: The solid black line is for simulation results and the red dotted line for the measured results.

The proposed antenna array is fabricated, as seen in Figure 11a, and validated experimentally. The obtained results from the numerical analysis are compared to the experimental measurements. As seen in Figure 11b,c, the measured S_11_ and gain spectra are presented. It is found that the experimental results agree very well with those obtained from CST MWS. The other relative measurements of the S-parameters in terms of S_12_, S_13_, and S_14_ spectra are seen in Figure 11d–f. It is validated that the maximum coupling is about −20 dB after the solar panel introduction, with excellent gain and bandwidth enhancements.

B. Correlation factors

**Figure 12:**
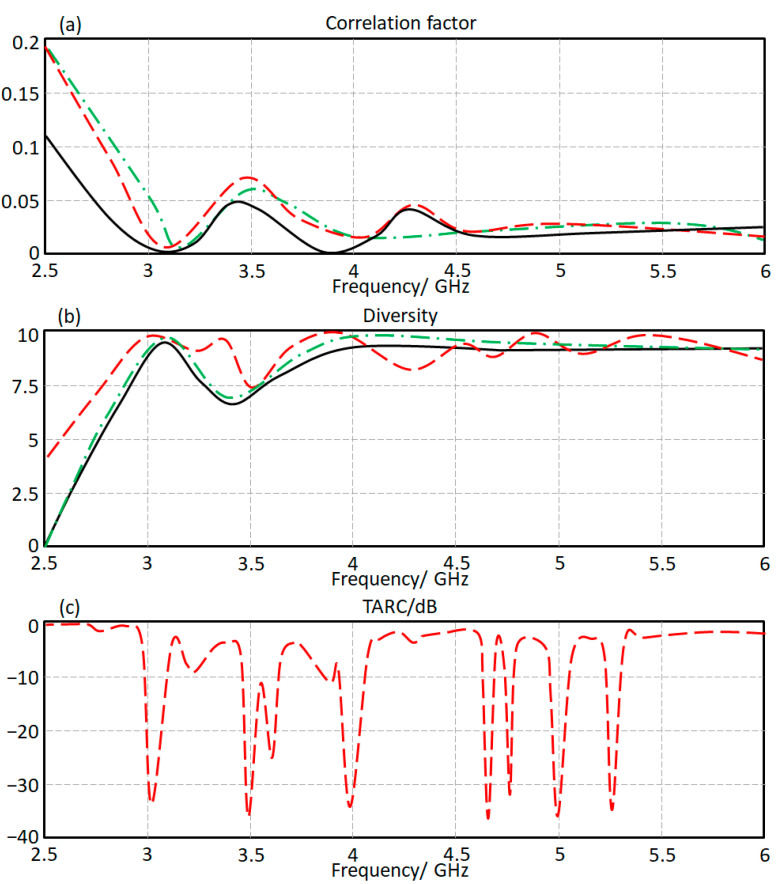
Antenna array performance in terms of: (**a**) correlation, (**b**) diversity, and (**c**) TARC.

Finally, the envelope correlation spectra are evaluated from the measured S-parameters [18] based on correlation, diversity, and TARC. In Figure 12, the correlation, diversity, and TARC spectra are measured and presented for the proposed antenna array. It is found that the correlation factor at the frequency of interest in terms of S_12_, S_13_, and S_14_ are found below 7%, as seen in Figure 12a. Whereas, the diversity is found to be almost about 9.7, as presented in Figure 12b. The TARC spectrum, as seen in Figure 12c, is found at about −24 dB at the frequency band of interest. Such results show the capability of the proposed antenna to reduce the correlation factor between the antenna elements.

C. Antenna Radiation Patterns

**Figure 13:**
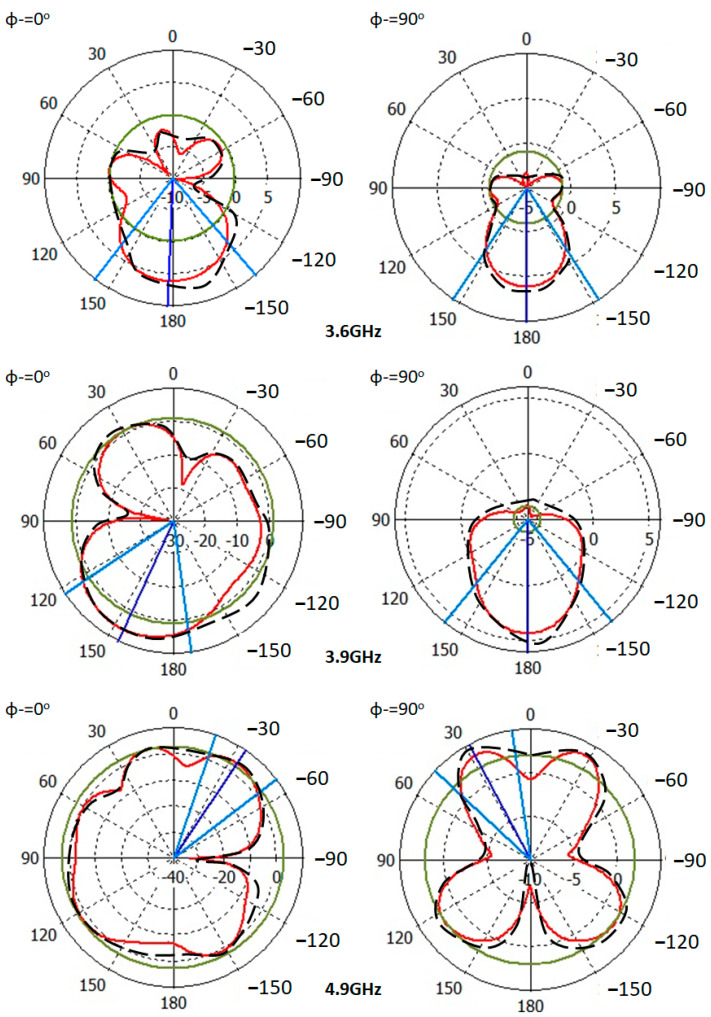
Antenna radiation patterns after the solar panel introduction. Note: The discrete black line is for simulation results and the red line for the measured results.

The proposed antenna radiation patterns are measured at 3.6 GHz, 3.9 GHz, and 4.9 GHz, as shown in Figure 13. It is found that the proposed antenna provides a significant enhancement in the directivity. The measured and simulated results agree well with each other.

D. Solar Energy Harvesting

**Figure 14:**
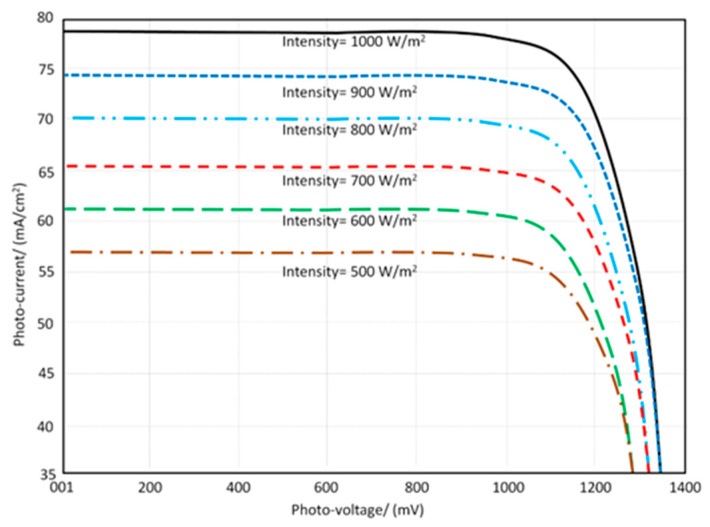
Solar panel performance after introducing the proposed antenna structure.

The planned antenna is put to the test in conjunction with a solar panel. As a result, before and after the solar panel integration, the measured I–V characteristics are given in Figure 14. The I–V characteristics are not greatly impacted after and before the solar panel integration, since the solar panels are placed properly to the antenna array.

After the planned antenna is installed, the I-V characteristics of the solar panels are measured. As a result, minimal effects on solar panel performance are discovered. This is due to the antenna array construction perpendicular on the solar panel. The solar energy is defined in the photo-current and photo-voltage curve at various light intensities by adjusting the value from 500 W/m^2^ to 1000 W/m^2^, as shown in Figure 14.

## 8. Conclusions

The proposed antenna array is structured from four antenna elements based on CRLH and open T-stub to maintain low coupling effects between the adjacent antennas. Therefore, the maximum coupling is found to be not more than −20 dB over the entire frequency band of interest. The proposed antenna radiation pattern is controlled with two LDRs to be changed from ±30°, ±40°, and ±45° at 3.6 GHz, 3.9 GHz, and 4.9 GHz, respectively. The antenna gain is found to be moderate with the range of 3.2 dBi to 6.9 dBi. Finally, the antenna provides a low correlation factor due to the high radiation diversity, which is achieved from the CRLH and open T-stub introductions. The proposed antenna array is integrated with solar cells for self-power applications in a 5G modern wireless communication system. The solar panel would be located perpendicular to the antenna array axis to maintain the antenna array performance. In future work, this antenna array will be integrated into radio frequency energy harvesting to offer efficient power for mobile and biomedical self-powered wireless devices.

## Figures and Tables

**Table 1:** Radiation properties with changing the LDR switching.

LDR_1_	LDR_2_	Frequency (GHz)	Gain (dBi)	Main Lobe Direction (Degree)
OFF	OFF	3.6	3.6	30°
		3.9	3.2	40°
		4.9	3.5	45°
OFF	ON	3.6	3.4	15°
		3.9	3.2	20°
		4.9	3.3	25°
ON	OFF	3.6	3.5	−15°
		3.9	3.5	−20°
		4.9	3.3	−25°
ON	ON	3.6	3.5	−30°
		3.9	6.9	−40°
		4.9	3.4	−45°

**Table 2:** Antenna performance with respect to the published results.

References	Antenna Array Size	Frequency Bandwidth/GHz	Maximum Gain/dBi	Coupling/dB	Separation Distance	Reconfiguration
[1]	0.3 λ × 0.224 λ	2.8 (S_11_ ≤ −10)	2.1	−15	λ/3.2	Not reconfigurable
[2]	0.682 λ × 0.682 λ	3.3–3.91 (S_11_ ≤ −10)	3.3	−16	λ/2.7	Not reconfigurable
[3]	1.8 λ × 1.8 λ	3.38–3.95 (S_11_ ≤ −10)	4.9	−10	λ/4	Not reconfigurable
[4]	0.778 λ × 0.567 λ	3.5 (S_11_ ≤ −10)	3.6	−18	λ/2.3	Not reconfigurable
[5]	0.8 λ × 0.799 λ	3.5 (S_11_ ≤ −10)	4.2	−13	λ/4.3	Not reconfigurable
[6]	1.55 λ × 1.55 λ	3.5 (S_11_ ≤ −10)	6.1	−19	λ/2.1	Radiation reconfiguration with PIN diodes
[7]	0.28 λ × 0.24 λ	2.8 (S_11_ ≤ −10)	4	−15	λ/3.4	Polarization reconfiguration with PIN diodes
[8]	0.967 λ × 2.127 λ	5.8 (S_11_ ≤ −10)	2.6	−10	λ/2.5	Not reconfigurable
[9]	0.5 λ × 0.5 λ	5.8 (S_11_ ≤ −10)	5.5	−14	λ/2.3	Not reconfigurable
[10]	0.967 λ × 0.967 λ	5.8 (S_11_ ≤ −10)	5.8	−15	λ/2.6	Not reconfigurable
[11]	1.77 λ × 0.9 λ	3.4–3.6 (S_11_ ≤ −10)	5.1	−15	λ/8.6	Not reconfigurable
[12]	1.7 λ × 0.9 λ	3.4–3.8, 5.15–5.925 (S_11_ ≤ −6)	6	−20	λ/10	Not reconfigurable
[13]	1.65 λ × 0.88 λ	3.3–3.8	5.3	−20	λ/28	Not reconfigurable
This work	0.744 λ × 0.48 λ	3.6, 3.9, 4.9 (S_11_ ≤ −10)	5	−20	λ/15	Radiation reconfiguration with LDRs

## Data Availability

Not applicable.
